# A Prospective cohort study investigates the health consequences and biomarkers in Iraqi radiation workers

**DOI:** 10.22038/aojnmb.2025.82707.1583

**Published:** 2025

**Authors:** Rasha Sabeeh Ahmed, Haidar Ahmed Shamran, Dunia Ali Shamsi

**Affiliations:** 1 Department of Physiology and Medical Physics, College of Medicine, Al-Nahrain University, Alkadhimiya, Baghdad, Iraq; 2 Medical research unit, College of Medicine, Al-Nahrain University, Alkadhimiya, Baghdad, Iraq

**Keywords:** Comet assay, MDA, Radiation hazard, Oxidative stress

## Abstract

**Objective(s):**

Objectives: Ionizing radiation has the potential to change the pattern of DNA methylation and can cause oxidative damage that may impact DNA. In this prospective cohort study, the effect of ionizing radiation on Iraqi radiation workers has been estimated by investigating the malondialdehyde levels, DNA methylation, DNA fragmentation, as well as the karyorrhectic, differentiated, and basal cells in buccal tissue.

**Methods:**

This work involved 80 participants, 40 hospital X-ray workers, and 40 control. Blood samples have been investigated using the NWLSS™ malondialdehyde assay, the gSYNC™ DNA extraction kit, and the comet assay.

**Results:**

The mean values of malondialdehyde level, DNA methylation, and DNA fragmentation (%DNA in tai), in workers were found to be 3.00±0.53, 51.63±26.44, and 26.73±12.67, respectively, while in the control were found to be 0.67±0.11, 13.25±11.30, and 9.09±11.96, respectively. In buccal tissue, the mean values of karyorrhectic cells, differentiated cells, late differentiated cells, and early differentiated cells in workers were found to be 7.70±4.64, 8.98±5.44, 14.74±5.25, and 18.50±6.40, respectively, while in control group were found to be 0.15±0.43, 0.20±0.41, 3.45±1.30, 10.78±1.80, respectively. The level of basal cells in buccal tissue was lower in workers (49.33±18.77) compared to the control group (85.75±2.19).

**Conclusions:**

The observed levels of biomarkers in radiation workers suggest adverse health consequences due to their occupational exposure to ionizing radiation. Controlling the exposure of hospital workers is the first step to improving the health of workers, thus decreasing economic and human costs.

## Introduction

 DNA damage due to multiple internal and external factors. The internal factors consist of oxidative stress, errors in DNA replication, and reactive oxygen species (ROS) that can result in strand breaks (1). External factors such as exposure to ionizing radiation, environmental pollutants, ultraviolet (UV) radiation, lifestyle habits like smoking, and chemicals further lead to DNA damage (2, 3). Ionizing radiation has been thoroughly documented to cause DNA damage; it is crucial to consider this factor when assessing genomic integrity in individuals who are exposed (4, 5).

 Radiation workers are among those occupationally exposed to Ionizing Radiation (IR) and are at increased risk of developing radiation-related health effects (6, 7). Ionizing radiation has sufficient energy to ionize atoms and molecules, leading to the production of reactive free radicals that can cause damage to cells and deoxyribonucleic acid DNA. Radiation workers may be exposed to IR during various procedures, including diagnostic imaging, interventional radiology, and radiation therapy. The risks associated with radiation exposure can be acute or chronic and may include skin damage, cataracts, and an increased risk of cancer (8, 9). Therefore, it is crucial to implement adequate radiation protection measures to minimize the risks associated with occupational exposure to IR among radiology workers. 

 DNA methylation, oxidative damage, and DNA fragmentation are three potential pathways that may link radiation exposure to DNA damage (10-12). DNA methylation is an epigenetic modification that regulates gene expression and is sensitive to environmental exposures (13, 14). In DNA methylation, a methyl group (CH3) is added to a cytosine (C) base within a specific dinucleotide sequence known as CpG dinucleotides (cytosine followed by a guanine nucleotide separated by a phosphate group), resulting in the formation of 5-methylcytosine. This process is catalyzed by a group of enzymes known as DNA methyltransferases.

 Adding a methyl group changes the chemical properties of the DNA molecule, making it less accessible to certain proteins that bind to DNA (15). This can have various consequences on gene expression, as proteins that bind to DNA are important regulators of gene transcription (the process by which the information encoded in DNA is converted into RNA molecules). Radiation can affect DNA methylation patterns by inducing changes in the activity of DNA methyltransferase enzymes or by altering the availability of the substrates necessary for DNA methylation (16, 17).

 Exposure to IR can also induce oxidative damage in cells by generating reactive oxygen species (ROS) through the radiolysis of water molecules (18, 19). ROS can react with DNA molecules, leading to the formation of DNA adducts and strand breaks. Ionizing radiation can directly interact with DNA molecules, causing ionization and excitation of atoms and molecules within the DNA structure. This process can generate free radicals, such as hydroxyl radicals (·OH), that can react with DNA bases, leading to the formation of various DNA adducts (20, 21). In addition to adduct formation, IR can also cause DNA strand breaks by inducing single-strand breaks (SSBs) and double-strand breaks (DSBs) (22, 23). SSBs can be caused by the direct action of radiation on the DNA backbone or by oxidative damage induced by radiation-generated ROS. DSBs can be caused by the interaction of IR with the DNA molecule, leading to clustered DNA damage sites. DNA fragmentation results from the physical breakage of DNA molecules due to radiation exposure. The resulting DNA damage can lead to a range of biological effects, including mutations, chromosomal aberrations, and cell death. In addition, oxidative damage induced by IR can affect various cellular processes, including DNA replication, transcription, and epigenetic modifications. Malondialdehyde (MDA) is a byproduct that results from the peroxidation of lipids, which is a form of oxidative stress caused by reactive oxygen species (ROS). The amount of MDA present can be used as an indicator of the extent of oxidative damage (24).

 Despite the potential importance of the mentioned pathways, few studies have investigated their role in mediating the effects of occupational radiation exposure on DNA damage in radiology workers in Iraq. The objective of this study is to evaluate the radiation-induced biomarkers, including DNA methylation status, oxidative damage, cell differentiation in buccal tissue, and DNA fragmentation, in radiation workers at some Iraqi hospitals. By investigating these pathways, we may better understand the mechanisms underlying radiation-induced DNA damage and identify potential targets for prevention strategies in occupational settings. This study is particularly important and novel because it is the first of its kind to combine these measures to assess the impact of radiation exposure on human health in Iraq, and no similar previous studies have been found in the literature.

## Methods

### Study Population and Sampling

 This study is a prospective cohort study and involved a group of 80 study participants, consisting of 40 radiation workers and 40 study participants who did not work in the radiation field. Table 1 provides a summary of the ages of the participants in each group, consisting of 12 males and 28 females. Blood samples were collected between May 2022 and July 2022 from workers at different X-ray rooms in Al Imamain Al-Kadimain Medical City, Baghdad Teaching Hospital, and Teaching Oncology Hospital in Baghdad/Iraq. Information collected included gender, age, duration of work in radiology, the type of device they worked with, smoking status, and history of chronic disease. The 40 samples were collected from radiation workers in X-ray, CT-scan, Mammogram, and fluoroscopy rooms (Table 2). The control samples were collected randomly from individuals who never worked in radiology departments and were matched by age and gender with the radiation workers, as summarized in Table 2. The years of service for radiation workers ranged from 0.3 to 24 years, with a mean±SD of 10.18±0.90 years.

**Table 1:** The minimum, maximum, and mean values of the ages of radiation workers and control groups in addition to grand total including data standard deviation (StdDev)

**Age (years)**	** Control**	**Worker**
Minimum	22	22
Maximum	56	54
Mean	36.23	35.85
StdDev	8.11	7.5

**Table 2:** The hospitals included in this study, the type of device used by the radiology worker, and the codes for both the hospital and the device

**Hospitals**	**Type of device**	**Code**
Al Imamain Al-Kadimain Medical City	Helical Ct-scan	KMC-HCT
Al Imamain Al-Kadimain Medical City	X-ray (room1)	KMC-XR1
Al Imamain Al-Kadimain Medical City	X-ray (room2)	KMC-XR2
Al Imamain Al-Kadimain Medical City	Mammogram	KMC-M
Baghdad Teaching Hospital	Helical Ct-scan	BTH-HCT
Baghdad Teaching Hospital	X-ray (room1)	BTH-XR1
Baghdad Teaching Hospital	X-ray (room2)	BTH-XR2
Baghdad Teaching Hospital (emergency)	Helical Ct-scan	BTHE-HCT
Baghdad Teaching Hospital (emergency)	X-ray room	BTHE-XR
Teaching oncology hospital	Ct-scan	TOH-CT
Baghdad Teaching Hospital (catheterization)	Fluoroscopy	BTHC-F

 The blood samples were obtained by venipuncture using a 5 mL syringe. The 5 mL sample was then separated into 3 mL of ethylene diamine tetraacetic acid (EDTA) tube and 2 mL of the gel tube to obtain plasma and serum. After leaving the gel tube samples to coagulate for 10 minutes at room temperature, they were spun in a centrifuge at 3000 rpm for 10 minutes. Using a micropipette, the serum and plasma were transferred to separate tubes and stored at a temperature of -20 °C until they were analyzed.

### Inclusion Criteria

 Healthcare professionals, such as radiologists, technicians, nurses, and service workers.

### Exclusion Criteria

 Individuals who had previously been diagnosed with chronic diseases such as liver and kidney diseases, coronary heart disease, stroke, myocardial infarction, diabetes mellitus, hypertension, and other serious organic disorders that could potentially interfere with the detection indices were not included in the study.

### Malondialdehyde (MDA) Level

 To determine the level of MDA, the malondialdehyde assay provided by Northwest Life Style Science Specialties (NWLSSTM) in the United States has been used. The NWLSS™ Malondialdehyde assay is used to quantify lipid peroxidation as the concentration of MDA (25). The NWK-MDA01 test uses the MDA and thiobarbituric acid (TBA) reaction, which forms a malondialdehyde-thiobarbituric acid adduct (MDA-TBA_2_) compound with a high absorption level at 532 nm.

 The NWLSS™ Malondialdehyde Assay protocol involves several steps. Firstly, Butylated Hydroxytoluene (BHT) Reagent (10 µL) is added to a microcentrifuge vial. Next, the blood sample (250 µL) is added, followed by the acid reagent (250 µL) and the TBA reagent (250 µL). 

 The solution is vortexed vigorously for 5 counts and then incubated for 60 minutes at 60 °C. The vial is centrifuged at 10,000 xg for 2-3 minutes, and the reaction mixture is transferred to a well plate. To prevent the oxidation of lipids, which can interfere with the accuracy of the sample processing and TBA reaction, the sample and reaction mixture are supplemented with BHT and EDTA (26). Moreover, the temperature of the reaction mixture is reduced to minimize the decomposition of lipid hydroperoxides. Since a significant portion of MDA is bound to proteins, mainly in the form of a Schiff base, the reaction pH is adjusted to facilitate the hydrolysis of MDA (27). In the end, the samples were added to a well plate containing the mixture. The plate was then placed into the GlowMax reader, which was calibrated to measure wavelengths between 400 and 700 nm. The device then displayed the concentration of MDA in mmol/L as the final result.

### DNA Methylation Level Measurements

 To extract genomic DNA from cells, the gSYNC™ DNA extraction kit uses a combination of proteinase K enzyme and chaotropic salt to lyse the cells and break down proteins. This allows the DNA to bind to a glass fiber matrix in the spin column while contaminants are removed using a washing buffer. Finally, the purified genomic DNA can be eluted using a low-salt elution buffer.

 The Blood Protocol Procedure involves preparing a sample of up to 200 μl of whole blood in a 1.5 ml microcentrifuge tube. The volume is adjusted to 200 μl with phosphate-buffered saline (PBS), and 20 μl of Proteinase K is added before mixing through pipetting. The mixture is then incubated at 60 ºC for 5 minutes. To lyse the cells, 200 μl of granular-sub-base buffer (GSB) is added, and the mixture is vigorously shaken to ensure thorough mixing. 

 The tube is then incubated at 60 ºC for 5 minutes, with inversion every 2 minutes. To bind the DNA, 200 μl of absolute ethanol is added to the sample lysate, and the mixture is immediately shaken vigorously for 10 seconds. 

 Next, a GS Column is placed in a 2 ml Collection Tube, and the entire mixture is transferred to the column. The column is then centrifuged at 14-16,000 ×g for 1 minute. Finally, the GS Column is transferred to a new 2 ml collection cube. To wash the DNA-bound column, 400 μl of W1 Buffer is added to the GS Column, and the column is centrifuged at 14-16,000 ×g for 30 seconds. The flow-through is then discarded, and the GS Column is returned to the 2 ml Collection Tube. Next, 600 μl of washing Buffer, which should contain added absolute ethanol, is added to the GS Column, followed by centrifugation at 14-16,000 ×g for 30 seconds. 

 The flow-through is again discarded, and the GS Column is returned to the 2 ml Collection Tube. Finally, the column is centrifuged for 3 minutes at 14-16,000 ×g to dry the column matrix. To elute the purified DNA, the dried GS Column is transferred to a clean 1.5 ml microcentrifuge tube. Then, 100 μl of pre-heated Elution Buffer, Tris-EDTA (TE) Buffer, or water is added to the center of the column matrix. The mixture is left to stand for at least 3 minutes to allow for complete absorption of the Elution Buffer, TE Buffer, or water. The column is then centrifuged at 14-16,000 ×g for 30 seconds to elute the purified DNA to be processed for real-time polymerase chain reaction (PCR) analysis to assess DNA methylation patterns. The Epitect Methylation PCR (QIAGEN/Germany) was used to amplify and quantify the purified DNA template in real-time, allowing for the precise measurement of the initial amount of DNA present in the sample.

### Investigating of the Mouth's Buccal

 Preparing buccal cells involves using a wooden spatula to collect cell samples from the inside of the mouth. The cells are then spread onto clean slides, with two slides being used for each sample from the right and left cheeks. To prevent bacteria from interfering with the analysis, a washing buffer with a pH of 7 is used. 

 The cell suspensions are then fixed and transferred onto the slides, which are left to air dry at room temperature. Eighty percent methanol was used during the fixation process. 

 A DNA-specific stain with a strong affinity for DNA is appropriate for distinguishing nuclear irregularities in shed cells from the lining of the mouth. The preferred staining technique is the modified Feulgen stain (also known as Feulgen-Schiff's fast green stain) because it reduces the likelihood of inaccurate results. Using this staining method, DNA structures such as nuclei and micronuclei (MN) can be observed under a microscope.

**Figure 1:**
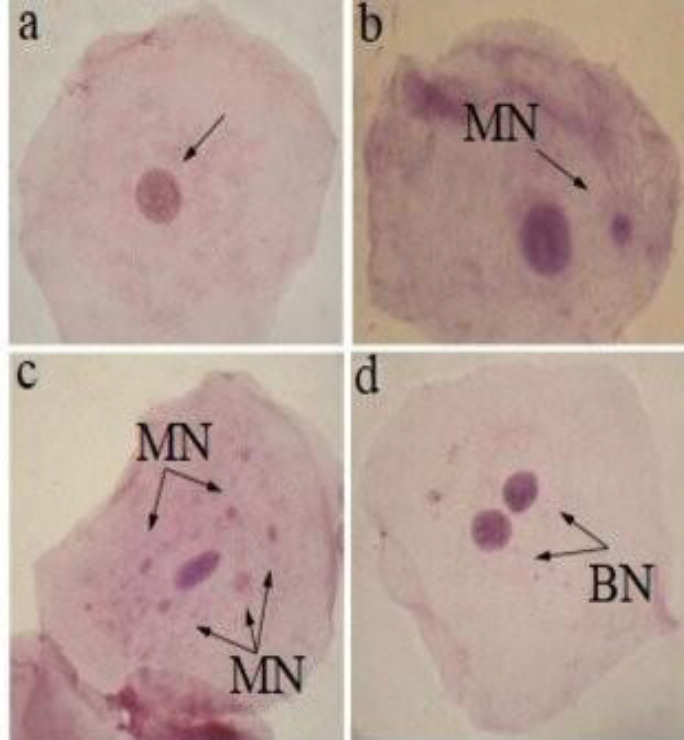
Exfoliated buccal epithelial cells with various nuclear anomalies at 1000× (**a**) normal cell; (**b**) micronuclei (MN); (**c**) five micronuclei (MN); (**d**) binucleated cell (BN)

**Figure 2:**
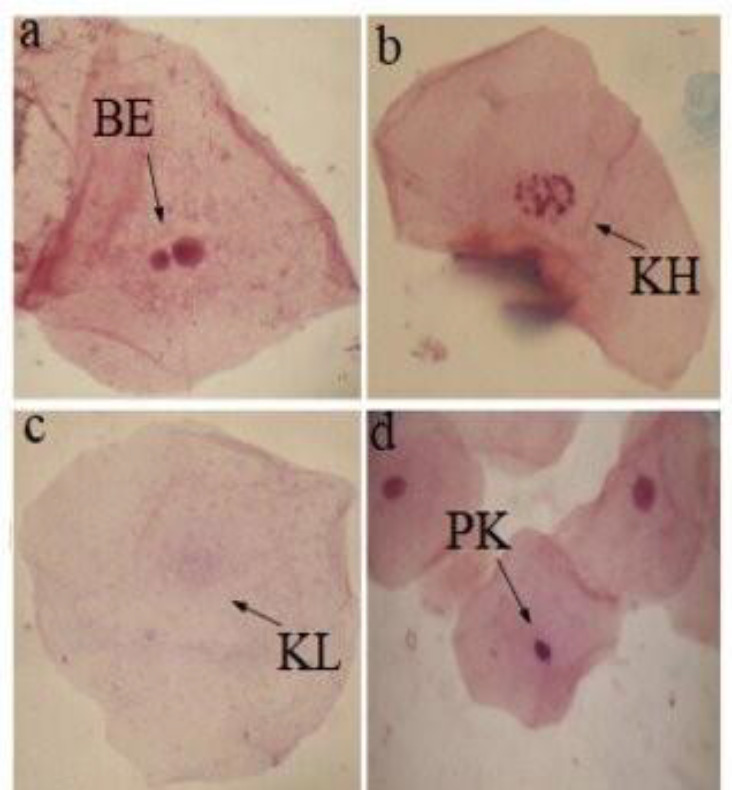
Exfoliated buccal epithelial cells with various nuclear anomalies at 1000× (**a**) broken egg (BE); (**b**) karyorrhexis (KH); (**c**) karyolysis (KL); (**d**) pyknosis (PK)

 A sample size of 1000-3000 cells per person was assessed to identify and categorize nuclear abnormalities, which is the most commonly employed method. Various nuclear anomalies were observed in comparison to a normal cell nucleus, as demonstrated in Figures 1 and 2.

### DNA Fragmentation Using Comet Assay

 The Comet assay, CometAssayTM Silver TREVIGEN/United States, which is also referred to as single-cell gel electrophoresis, is a highly sensitive method for assessing DNA damage in individual cells. The assay involves damaged or cleaved DNA fragments being denatured and migrating out of the cell during electrophoresis, which produces a distinctive "comet tail" formation. Conversely, undamaged DNA remains within the cell membrane, creating the "comet head".

 The assay protocol begins by adding 5 mL of diluted PBS to a 500 μL blood sample, followed by centrifugation at 2500 rpm for 10 minutes. The solution is partially removed, leaving a small amount at the end of the tube, and another 5 mL of diluted PBS is added to the remaining solution. The cells are then incubated in an incubator at 37 °C for 5 minutes and centrifuged again at 2500 rpm for 10 minutes. After removing the excess solution, 5 mL of diluted R-lysis solution is added to the cells and left for 5 minutes before being centrifuged again at 2500 rpm for 10 minutes. The cells are then left for 24 hours. Low melting (LM) Agarose is melted in boiling water for 5 minutes and then cooled in a 37 °C water bath for at least 20 minutes. 100 μL of LM Agarose and 100 μL of cells are mixed in a microtube and speared onto a slide. The slides are cooled in a deep freeze (-20 °C) for 20 minutes, immersed in prechilled Lysis Solution for two hours, and the excess buffer is removed before being immersed in freshly prepared Alkaline Unwinding Solution. The slides are then left in an alkali unwinding solution for 20 to 60 minutes, removed, taped gently to remove excess buffer, and washed by immersing in tris borate EDTA (TBE) buffer for 5 minutes. The slides are transferred to a horizontal electrophoresis apparatus and calibrated to 75 V and 300 mA for 30 minutes for each of the four slides. The slides are then dipped in 70% ethanol for 5 minutes, air-dried, and stained with diluted ethidium bromide for 30 minutes at room temperature. The intensity of staining can be visualized under a microscope using a 10× objective, and the reaction is stopped when comets are easily visible. The intensity of DNA content in the comet tail can be used for scoring. 

**Figure 3:**
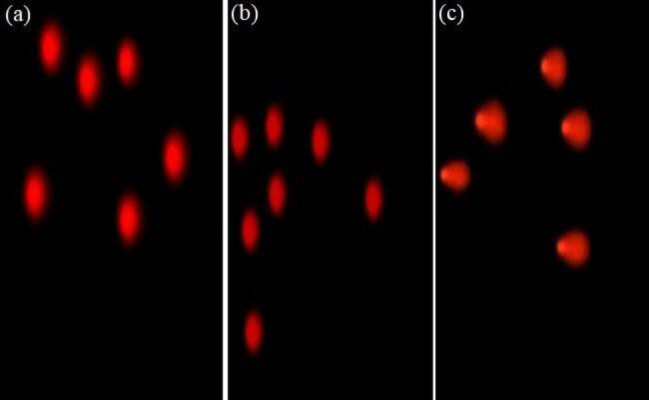
Comet score images indicating the comet length for some of the investigated samples (**a**, **b** for controls, and **c** for workers)

 It is important to use untreated cells as a control to establish the features of healthy cells. Scoring should be based on nominal, medium, or high intensity of tail DNA content. A minimum of 100 cells should be evaluated per sample. The Comet Score Application Program, also known as Comet Score, can measure various parameters including comet length, comet height, comet area, tail length, tail area in pixels, percentage of DNA in the head, percentage of DNA in the tail, and tail moment (Figure 3). These are the most frequently measured parameters. Tail length, %DNA in the tail, and tail moment are regarded as reliable indicators of DNA damage (28).

### Statistical analysis

 In this study, the data was inputted, verified, and assessed through IBM SPSS software version 26. To outline the characteristics of the data that was collected, mean descriptive statistics were used. To determine the significance among the groups within the study, several tests such as student t-test, and Paired sample t-test were utilized. The P-value that indicated the differences among the study’s groups was less than 0.05 in this investigation.

## Results

### The Malondialdehyde Levels

**Figure 4:**
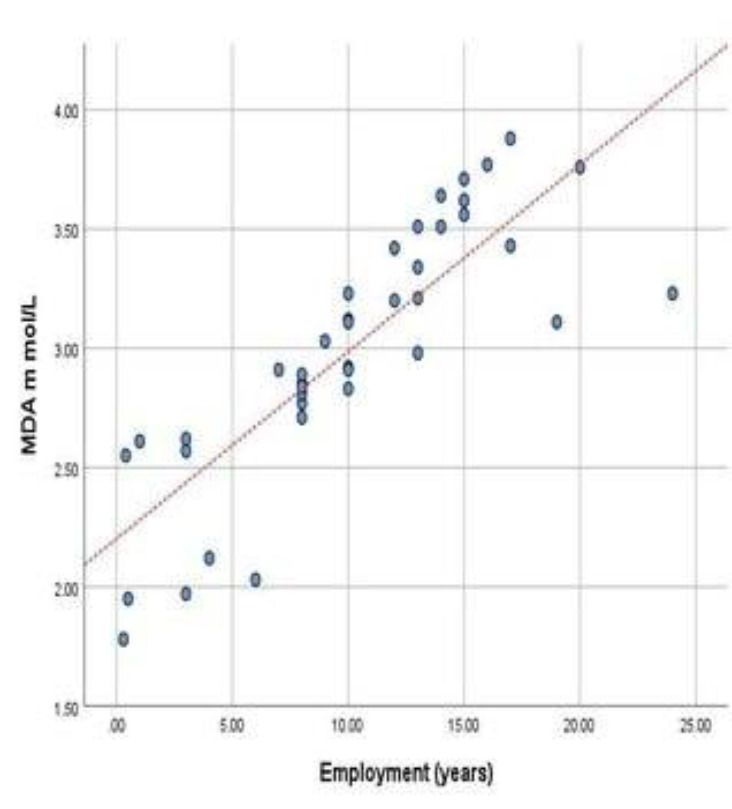
Line graph showing a significant positive correlation between the level of MDA and years of employment. (P value=0.000)

 The result of MDA assessments indicates a significant difference in MDA level between the workers (3.00±0.53) and control group (0.67± 0.11), with a P-value of 0.000. In other words, the outcomes revealed that the workers experienced more oxidative stress than the control group, which was significantly higher. Our findings indicated a substantial positive association (Pearson correlation=0.83) between the level of MDA and the years of employment, as seen in Figure 4.

### The DNA Methylation

 The outcomes revealed a noteworthy difference in DNA methylation levels between the radiation workers (51.63±26.44) and the control group (13.25±11.30), with a P-value of 0.000. Specifically, we observed a significant increase in DNA methylation levels among the 

radiation workers compared to the control group, which indicates that the DNA methylation effects may be radiation dosage-dependent.

**Figure 5:**
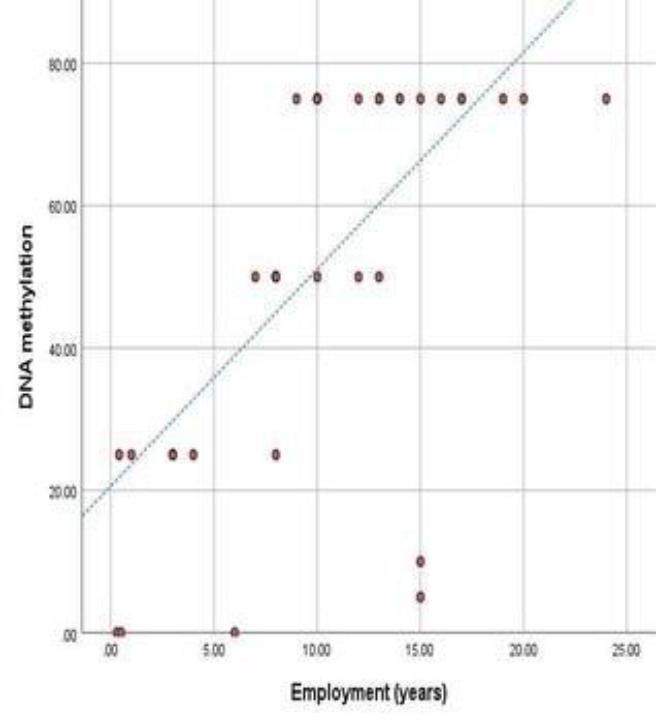
Line graph showing a significant positive correlation between DNA methylation and years of employment (P value=0.000)

Figure 5 shows a significant positive correlation (Pearson correlation=0.65) between the years of occupation and DNA methylation (P value=0.000).

### The Karyorrhectic, Differentiated, and Basal Cells in Buccal Tissue

**Figure 6:**
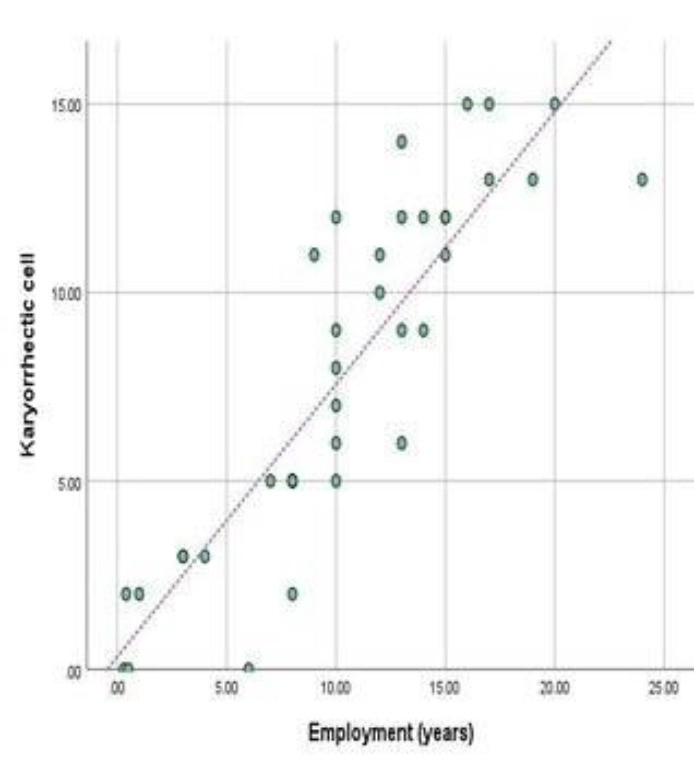
Line graph showing a significant positive correlation between karyorrhectic cells and years of employment (P value=0.000)

 The results in Table 3 indicate the frequency of karyorrhectic cells in buccal from the two groups: workers with an average of 7.70±4.64 and a control group with an average of 0.15±0.43. Our findings indicate a significant correlation between the occurrences of karyo-rrhectic cells in buccal tissue from both worker and control groups, with a p-value of 0.000. Our results suggest a positive linear relationship (Pearson correlation=0.88) between the number of years an individual has been employed and the presence of karyorrhectic cells, as seen in Figure 6.

**Table 3:** The mean values of karyorrhectic, differentiated, and basal cells in buccal tissue ± standard deviation in worker and control groups

		**Mean** ± ** Std**	**P value**
**Karyorrhectic cells**	worker	7.70±4.64	0.000
	control	0.15±0.43	0.000
**Differentiated cells**	worker	8.98±5.44	0.000
	control	0.20±0.41	0.000
**Late differentiated cells**	worker	14.74±5.25	0.000
	control	3.45±1.30	0.000
**Early differentiated cells**	worker	18.50±6.40	0.000
	control	10.78±1.80	0.000
**Basal cells**	worker	49.33±18.77	0.000
	control	85.75±2.19	0.000

**Figure 7:**
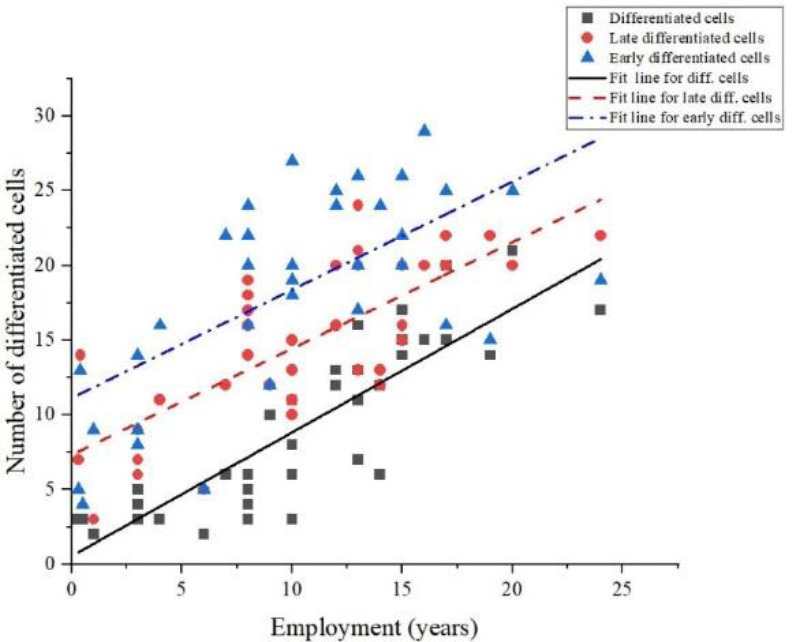
Line graph depicting a strong positive correlation (P-value of 0.000) between the number of differentiated cells with nuclear buds, the number of early differentiated cells with micronuclei, and the number of late differentiated cells with micronuclei with the number of years of employment

Table 3 shows the significant correlation (P value=0.000) between the differentiated cells in the buccal tissue of radiation workers and the control groups. The results of our study indicate that there is a significant positive correlation (Pearson correlation=0.86, 0.64, and 0.75 for differentiated, early differentiated, and late differentiated cells, respectively) between years of employment in a radiation field and the presence of differentiated cells in buccal tissue, as seen in Figure 7.

**Figure 8:**
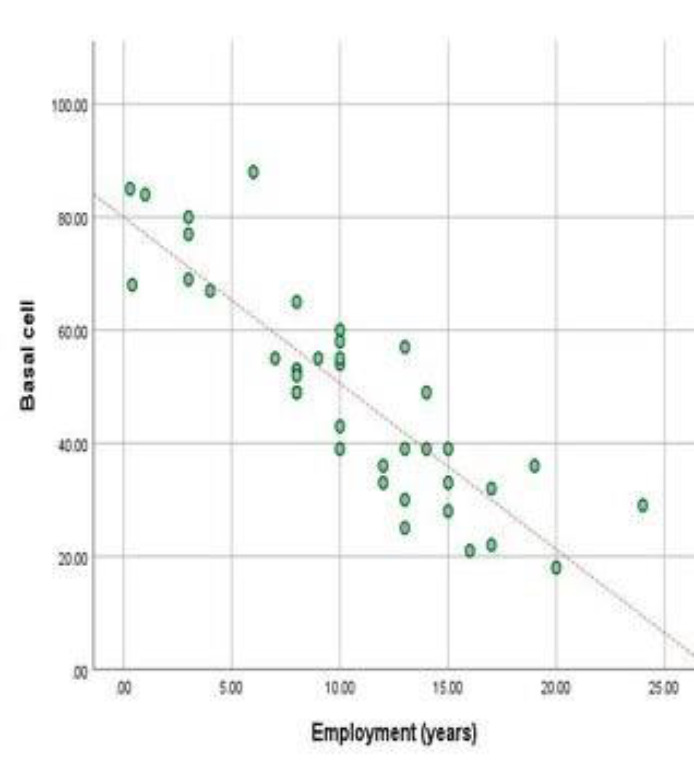
Line graph showing a significant negative correlation between basal cells and years of employment (P value=0.000)

 The obtained result suggests a significant correlation (P value=0.000) in the value of basal cells in a radiology worker compared to a control individual. The radiology worker has a value of basal cells of 49.33, while the control individual has 85.75. Our results revealed that there is a strong negative correlation (Pearson correlation= -0.86) between employment years and the value of basal cells, as seen in Figure 8.

### DNA Fragmentation

Table 4 illustrates the correlation between the mean values of tail length, tail area, tail intensity, %DNA in the tail, and tail moment for the exposed and the control groups.

**Table 4:** The obtained results of the comet assay on the blood of radiation workers and control groups

		**Mean±Std**	**P value**
**Tail length (px)**	Worker	8.58±5.6	0.000
	Control	2.08±3.17	0.000
**Tail area (px)**	Worker	196.05±146.96	0.000
	Control	45.28±75.1	0.000
**Tail intensity**	Worker	18163.23±13404.38	0.000
	Control	6980.3±9405.67	0.000
**%DNA in tail**	Worker	26.73±12.67	0.000
	Control	9.09±11.96	0.000
**Tail moment**	Worker	2.62±2.33	0.000
	Control	0.44±1.07	0.000

**Figure 9:**
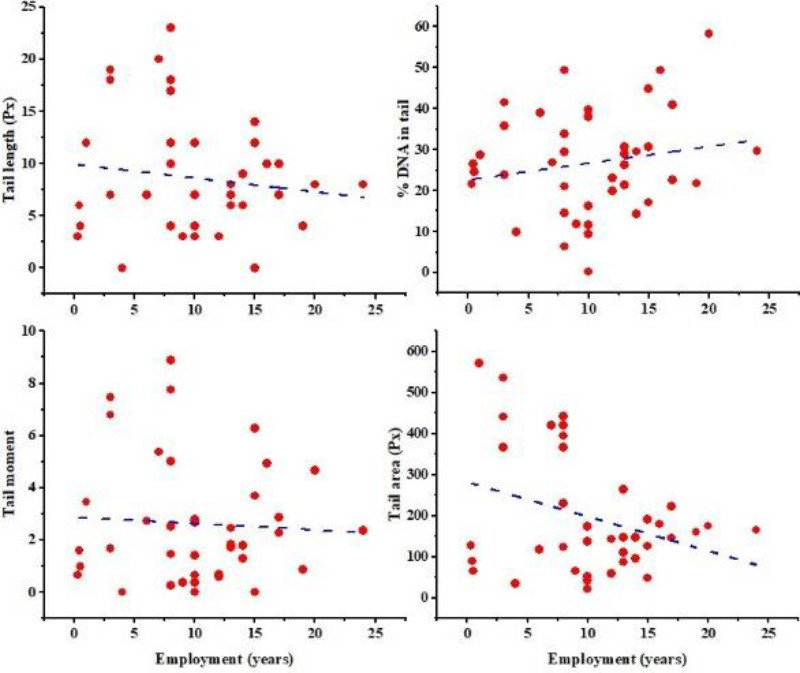
Line graph indicates that there is no significant correlation between the length of the tail, the percentage of DNA in the tail, the tail moment, the tail area, and the duration of employment (P value=0.000)

The results indicate a significant correlation (P value=0.000) in tail length between the worker and the control groups (8.58±5.6 and 2.08±3.17 Px, respectively). The tail area showed a notable association between the worker and the control groups, with a P value of 0.000. The tail area of the worker group was measured to be 196.05±146.96 Px, while the control group had a tail area of 45.28±75.1 Px. Our investigation reveals a significant difference (P value=0.000) in % DNA in the tail between the worker and the control group (26.73±12.67 and 9.09±11.96 Px, respectively). Our study demonstrates a significant correlation (P value=0.000) in the tail moment between the worker and the control groups (2.62±2.33 and 0.44±1.07, respectively), which suggests that DNA damage may be present. This study revealed that there was no significant correlation between tail area, tail moment, tail length, and %DNA in participants who had been exposed to radiation and years of service (P value= 0.04, 0.71, 0.41, and 0.26, respectively), as indicated by the Pearson correlation coefficients of -0.32, -0.06, -0.13, and 0.18, respectively (Figure 9). Similar results have been reported in other studies (29, 30).

## Discussion

 Oxidative stress occurs when the body's antioxidant defense system is overwhelmed by reactive oxygen species (ROS) and free radicals. Measuring the level of MDA in the blood samples can provide insight into the degree of oxidative stress in the body. This study indicates that workers experience significantly higher oxidative stress than the control group. This difference can be attributed to exposure to X-ray radiation. Other factors, including lifestyle, use of personal protective equipment, and genetic susceptibility, may influence these markers. Research consistently indicates that workers in the X-ray department exhibit higher levels of oxidative stress than the control groups. El-Benhawy and colleagues (31) found that study participants who were exposed to radiation had notably higher levels of oxygen-free radicals than those who were not exposed. Additionally, Kłucinski and his team (32) reported that persistent exposure to low levels of IR and chronic oxidative stress could decrease the antioxidant levels in workers. A study conducted by Bolbol and his colleagues (33) demonstrated that the exposed group had considerably elevated levels of MDA compared to the control group. According to Gao et al. (34), their analysis of MDA levels revealed a notable rise in hospital staff compared to the control group. Conversely, some previous studies have found that the levels of free radicals, MDA, were significantly higher in radiation-exposed workers than the control group, which contrasts with our findings (35, 36). Furthermore, our results indicated a significant positive Pearson correlation=0.83 between MDA and the years of employment. Workers with long tenures must follow strict radiation safety standard protocols, including limiting unnecessary exposure, employing protective shielding equipment, and changing job assignments to reduce long-term exposure.

 The significance of DNA methylation can be observed in various developmental processes, as well as in the imprinting of cell proliferation and maintaining genome stability (37). Recent studies have shown that IR exposure can alter DNA methylation patterns, including gene-specific DNA methylation and methylation of repetitive elements (16). It is unclear what the threshold dose of IR is for DNA methylation. The International Commission on Radiological Protection (ICRP) allows for an occupational exposure dose of up to 20 mSv per year. Still, no evidence suggests that this permitted dose impacts biochemical processes like DNA methylation. As shown in the results, workers subjected to ionizing radiation, like those working in X-ray departments, show markedly high DNA methylation levels compared to the control group, indicating a possible epigenetic response to long-term radiation exposure. 

 Ionizing radiation can cause oxidative stress and DNA damage, triggering epigenetic modifications with increased methylation at particular gene locations engaged in DNA repair, apoptosis, and tumor suppression. This may affect genomic stability and elevate the risk of cancerous diseases. According to Chen et al.'s study, their findings indicate that occupational irradiation has a particular influence on DNA methylation in interventional radiation physicians compared to the control group (38). 

 Wdowiak et al. (39) conducted a study to evaluate the health effects of exposure to IR from artificial sources, including medical ones. They discovered that 30 Gy is the minimum dose that can cause detectable DNA damage. In males exposed to IR, the genetic material in the sperm of these males showed higher fragmentation and methylation of DNA. These findings suggest that prolonged exposure to IR can lead to changes in DNA methylation patterns, which in turn may increase the risk of developing certain diseases, such as cancer. In this study, the noted rise in DNA methylation aligns with earlier research indicating that prolonged radiation exposure can alter the epigenetic profile, acting as a biomarker for biological effects caused by radiation.

 Karyorrhectic cells refer to cells that have undergone a process called karyorrhexis, which is the fragmentation of the nucleus of a cell. The presence of karyorrhectic cells in buccal tissue can indicate cell damage or stress. Additionally, karyorrhectic cells in buccal tissue can provide important information about the individual’s health. Karyorrhectic cells in worker buccal are approximately 51-fold greater than that in control. The results are not surprising as IR is known to cause single-strand and double-strand breaks in the DNA, which can trigger a response from the cell to try to repair the damage. However, if the damage is severe, the cell may undergo programmed cell death (apoptosis) or become damaged beyond repair, leading to karyorrhexis and the formation of karyorrhectic cells. Exposure to high levels of IR can cause significant damage to buccal cells and increase the risk of developing karyorrhectic cells. The effects of IR on buccal cells can depend on the duration of exposure.

 Differentiated cells in buccal tissue are cells that have reached their final stage of development and have stopped dividing. These cells have specialized structures and functions that are important for maintaining the health and function of the buccal tissue. Late differentiated cells in buccal tissue are fully mature cells and have completed their specialized functions. They are highly specialized and have lost their ability to divide further. Early differentiated cells in buccal tissue are cells that are in the process of maturing and acquiring specialized structures and functions. The results indicate that IR can induce differentiation in buccal tissue. This is because IR can damage stem cells in the buccal tissue, which can trigger the remaining cells to differentiate to repair the damage. This can lead to a loss of function or altered function in the differentiated cells, which can harm the overall health of the individual. The significant positive correlation between the years of employment in a radiation field and the presence of differentiated cells in buccal tissue suggests that prolonged exposure to IR can have a cumulative effect on differentiated cells, potentially leading to DNA damage and alterations in cell function.

 Basal cells are also found in the buccal mucosa, which is important in maintaining tissue integrity and renewal. The buccal mucosa is a stratified squamous epithelium composed of multiple layers of cells. The basal layer is the deepest layer of the epithelium, and it contains basal cells. Basal cells in buccal tissue divide and differentiate into other cell types, such as keratinocytes, which help to form the outer layers of the epithelium. In addition to their role in tissue renewal, basal cells in buccal tissue can also be a target for genetic mutations that can lead to the development of cancer. For example, oral squamous cell carcinoma is a cancer that can arise from basal cells in the buccal mucosa. 

 When IR penetrates tissues such as the buccal mucosa, it can cause damage to the DNA of basal cells. This damage can range from single-strand breaks to double-strand breaks in the DNA molecule and can lead to mutations that may result in cell death, abnormal cell growth, or cancer. The severity of the damage depends on factors such as the intensity of the radiation, the duration of exposure, and the sensitivity of the cells to radiation.

 IR can affect the ability of basal cells to divide and differentiate. This can decrease the number of basal cells, which can impair the ability of the buccal mucosa to renew and repair itself. The effects of IR on basal cells in the buccal mucosa can lead to adverse health effects, including oral cancer. Long-term exposure to IR, such as that experienced by study participants working in the radiation field, can increase the risk of oral cancer. The lower basal cell counts in radiation workers compared to the control group indicate that the radiology workers have experienced some degree of damage to their basal cells as a result of their exposure to radiation in the workplace. However, it is important to note that a single result alone does not provide sufficient information to draw definitive conclusions about the health of an individual. Factors such as the duration of radiation exposure can influence the health of an individual's basal cells.

 The comet assay is commonly utilized to assess the extent of DNA damage, which can be used as an indicator of susceptibility to radiation and identify potential health hazards in populations exposed to radiation. Although most instances of DNA damage are repaired within a short time following exposure, some damage remains unrepaired or is improperly repaired. In some cases, both the occurrence of mutations and inadequate repair of DNA damage may provide an early indication of the likelihood of developing cancer.

 Comet assay parameters like tail length, %DNA in the tail, and tail moment can quantitatively evaluate DNA damage. A longer tail length signifies a higher level of DNA fragmentation. The %DNA in the tail indicates the amount of fragmented DNA concerning the total cellular DNA, with higher values indicating more significant DNA damage. Tail moment, assessed as the product of the tail %DNA and tail length, is a more precise indicator of DNA damage compared to either parameter alone, since it considered both the extent and intensity of the tail. Considering that occupational radiation exposure may lead to genomic instability, evaluating these parameters offers essential insights into the genotoxic effects caused by radiation.

 Our research on DNA fragmentation revealed a notable variation in tail length, tail area, %DNA in the tail, and tail moment between radiation workers and the control group, showing elevated DNA damage in the worker group. These outcomes reveal that occupational radiation exposure increases DNA strand breaks, as shown in the comet assay parameters. Nonetheless, no notable correlation was found between %DNA, tail moment, tail area, tail length, and years of work, indicating that the level of DNA damage may be affected more by person susceptibility, the dose of radiation, and repair mechanisms instead of total exposure duration.

 Although radiation exposure can cause DNA damage, it does not always result in disease. The degree of damage and the biological effects it causes mainly rely on individual DNA repair capacity, which differs from person to person and seems to be elevated in radiation-exposed workers as a result of extended low-dose exposure. This adaptation might be related to radiation hormesis. This occurrence where low-dose radiation exposure activates cellular defense mechanisms, such as improved DNA repair, antioxidant activity, and apoptosis of damaged cells (40). Research indicates that long-term exposure to low doses of radiation can trigger protective mechanisms that lower the long-term genomic instability risk (41). 

 Nonetheless, the equilibrium between helpful adaptive responses and possible negative effects continues to be a topic of active discussion. Additional research is essential to assess how radiation exposure transitions from being adaptive to detrimental, specifically in occupational settings.

## Conclusion

 Based on the results, it can be concluded that the high levels of DNA methylation, malondialdehyde levels, karyorrhectic, differentiated, basal cells in buccal tissue, and DNA fragmentation observed in radiation workers compared to the control group suggest that they may be experiencing adverse health effects due to their occupational exposure to IR. The significant correlation between years of occupancy and all measured parameters further supports the notion that occupational exposure to IR may be responsible for the observed effects. 

 It is worth noting that the negative correlation between years of service and the tail area, tail moment, tail length, and %DNA in participants exposed to radiation may be due to comet assay sensitivity to oxidative damage rather than radiation exposure. In light of these findings, it is recommended that measures be taken to reduce the level of occupational exposure to IR in these hospitals to minimize the potential health risks to workers. Regular monitoring of the health status of hospital workers may also be necessary to detect any adverse effects of their exposure to IR. This study represents an important step towards a better understanding of the health risks associated with occupational exposure to IR in hospitals and underscores the importance of prioritizing the safety of hospital workers.
